# Dispersal of Epithelium-Associated Pseudomonas aeruginosa Biofilms

**DOI:** 10.1128/mSphere.00630-20

**Published:** 2020-07-15

**Authors:** Anna C. Zemke, Emily J. D’Amico, Emily C. Snell, Angela M. Torres, Naomi Kasturiarachi, Jennifer M. Bomberger

**Affiliations:** a Division of Pulmonary, Allergy and Critical Care Medicine, Department of Medicine, University of Pittsburgh, Pittsburgh, Pennsylvania, USA; b Department of Microbiology and Molecular Genetics, University of Pittsburgh, Pittsburgh, Pennsylvania, USA; University of Kentucky

**Keywords:** *Pseudomonas aeruginosa*, biofilm, cyclic-di-GMP, cystic fibrosis, dispersal, dispersion

## Abstract

Pseudomonas aeruginosa grows in highly antibiotic-tolerant biofilms during chronic airway infections. Dispersal of bacteria from biofilms may restore antibiotic susceptibility or improve host clearance. We describe models to study biofilm dispersal in the nutritionally complex environment of the human airway. P. aeruginosa was cocultured in the apical surface of airway epithelial cells (AECs) in a perfusion chamber. Dispersal, triggered by sodium nitrite, a nitric oxide (NO) donor, was tracked by live cell microscopy. Next, a static model was developed in which biofilms were grown on polarized AECs without flow. We observed that NO-triggered biofilm dispersal was an energy-dependent process. From the existing literature, NO-mediated biofilm dispersal is regulated by DipA, NbdA, RbdA, and MucR. Interestingly, altered signaling pathways appear to be used in this model, as deletion of these genes failed to block NO-induced biofilm dispersal. Similar results were observed using biofilms grown in an abiotic model on glass with iron-supplemented cell culture medium. In cystic fibrosis, airway mucus contributes to the growth environment, and a wide range of bacterial phenotypes are observed; therefore, we tested biofilm dispersal in a panel of late cystic fibrosis clinical isolates cocultured in the mucus overlying primary human AECs. Finally, we examined dispersal in combination with the clinically used antibiotics ciprofloxacin, aztreonam and tobramycin. In summary, we have validated models to study biofilm dispersal in environments that recapitulate key features of the airway and identified combinations of currently used antibiotics that may enhance the therapeutic effect of biofilm dispersal.

**IMPORTANCE** During chronic lung infections, Pseudomonas aeruginosa grows in highly antibiotic-tolerant communities called biofilms that are difficult for the host to clear. We have developed models for studying P. aeruginosa biofilm dispersal in environments that replicate key features of the airway. We found that mechanisms of biofilm dispersal in these models may employ alternative or additional signaling mechanisms, highlighting the importance of the growth environment in dispersal events. We have adapted the models to accommodate apical fluid flow, bacterial clinical isolates, antibiotics, and primary human airway epithelial cells, all of which are relevant to understanding bacterial behaviors in the context of human disease. We also examined dispersal agents in combination with commonly used antipseudomonal antibiotics and saw improved clearance when nitrite was combined with the antibiotic aztreonam.

## Abstract (precis)

During chronic lung infections, Pseudomonas aeruginosa grows in highly antibiotic-tolerant communities called biofilms that are difficult for the host to clear. We have developed models for studying P. aeruginosa biofilm dispersal in environments that replicate key features of the airway. We found that mechanisms of biofilm dispersal in these models may employ alternative or additional signaling mechanisms, highlighting the importance of the growth environment in dispersal events. We have adapted the models to accommodate apical fluid flow, bacterial clinical isolates, antibiotics, and primary human airway epithelial cells, all of which are relevant to understanding bacterial behaviors in the context of human disease. We also examined dispersal agents in combination with commonly used antipseudomonal antibiotics and saw improved clearance when nitrite was combined with the antibiotic aztreonam.

## INTRODUCTION

Pseudomonas aeruginosa is a common cause of chronic airway infections in cystic fibrosis (CF), chronic obstructive pulmonary disease, and non-CF bronchiectasis and following lung transplant (1–3). During chronic airway infections, the organism grows in biofilm communities either in close association with the epithelial surface or suspended in the airway mucus (4, 5). Communal growth in the airway is accompanied by extremely high antibiotic tolerance, making long-term eradication of well-established airway infections difficult (6–8). The switch from motile lifestyle to biofilm growth is highly regulated and influenced by diverse environmental factors (9–12). Dispersal from biofilms has been proposed as a therapeutic adjunct with the theoretical potential to improve antibiotic susceptibility and host phagocytosis (13–16). Given the many influences of the environment on bacterial behaviors, we sought to study biofilm dispersal in clinically relevant airway environments and, in this context, the ability of biofilm dispersal to improve the antimicrobial therapies used to treat chronic infections.

The switch between biofilm and planktonic lifestyles has been elegantly studied both in flow cells in which bacterial communities are grown on abiotic surfaces and in static systems (17, 18). Dispersal of P. aeruginosa biofilms has been reported in response to many triggers, including nitric oxide, 2-cis decanoic acid, changes in carbon source, carbon starvation, divalent cation chelation, bacteriophage-mediated lysis, and hyperthermia matrix-degrading enzymes (summarized in reference 12; primary sources, references 9, 10, and 19–26). Using these triggers, cyclic-di-GMP signaling has been defined as the core second messenger regulating biofilm dispersal and the resumption of a motile lifestyle (19, 20, 27, 28). The P. aeruginosa lab strains PAO1 and PA14 express up to 41 enzymes regulating the concentration of cyclic-di-GMP (29, 30). Individual phosphodiesterases (PDEs) and diguanylyl cyclases (DGCs) have been shown to play distinct roles in swarming motility, surface-sensing behaviors, antimicrobial tolerance, exopolysaccharide production, and biofilm dispersal (20, 31–33). Specifically, biofilm dispersal in response to nitric oxide is dependent on the presence of DipA and NbdA in some systems (20, 34). The current understanding of NO-induced biofilm dispersal is that it requires the enzymatic activity of DipA and NbdA with a subsequent drop in cyclic-di-GMP levels. However, the coordinated behavior of these enzymes in more complex experimental systems remains to be elucidated and is likely context specific.

The bacterial physiology of dispersal in the nutritionally complex airway environment is poorly understood. The presence of airway epithelial cells can influence key bacterial behaviors. For example, biofilm growth in the presence of airway epithelial cells, compared to on abiotic surfaces, greatly increases antimicrobial tolerance and alters the transcriptional response to antibiotics (7, 35). As another key example, the nutritional environment modulates cyclic-di-GMP signaling and the downstream behavior of swarming, again reflecting the importance of the environment to bacterial behaviors (31). Finally, recent work has shown that the role of a specific phosphodiesterase in motility varies widely with medium type despite similar cyclic-di-GMP levels, showing that additional layers of regulation exist for some motility behaviors beyond absolute cyclic-di-GMP level (36). Because biofilm dispersal is proposed as a therapeutic adjunct in chronic airway infections, it is particularly important to understand whether and how mechanisms of dispersal in the airway environment resemble those mechanisms described in abiotic systems. The purpose of the current study was to establish a biofilm dispersal model that simulates chronic airway infections. We developed models in which P. aeruginosa is cocultured with airway epithelial cells to form bacterial aggregates, and then dispersal is induced and measured. Using these models, we were able to develop dispersal assays for clinical bacterial isolates, study dispersal in the presence of a mucus-secreting primary human airway epithelium, combine dispersal agents with commonly used antipseudomonal antibiotics, and study dispersal signaling. The models extend our understanding of communal bacterial behavior in the airway environment.

## RESULTS

### Development of biofilm dispersal models.

**FIG 1:**
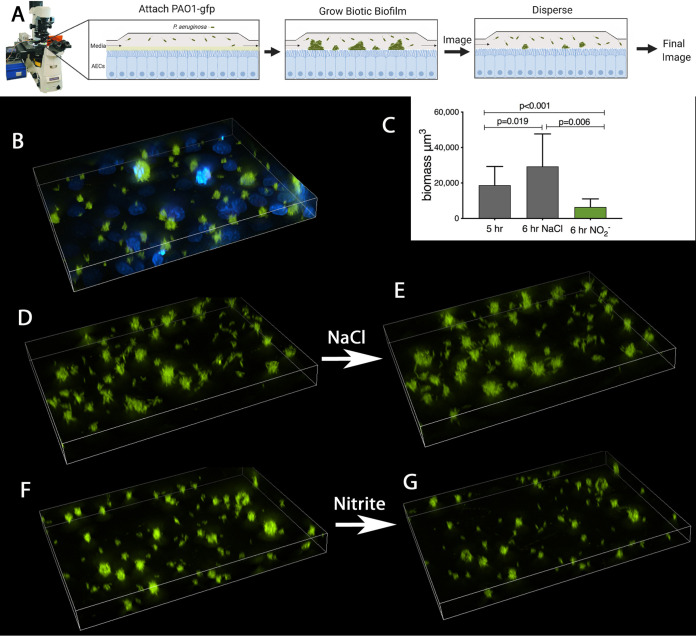
Biotic biofilm dispersal in flow cells. (A) CFBE41o- airway epithelial cells are grown on glass coverslips and placed in perfusion chambers. The chambers are inoculated with PAO1-gfp (green). Bacteria attach for 60 min, and then the biofilm is grown for 4 more h. Then, from hours 5 to 6, the coculture is treated with NaCl (tonicity control) or 15 mM sodium nitrite. Images of identical points are taken at hours 5 and 6. (B) Volumetric projection of representative coculture after 6 h taken at ×40 magnification. Blue (DAPI) stains the epithelial nuclei beneath the PAO1 (green) biofilms. (C) Quantification of biomass before (hour 5) and after (hour 6) treatment. (D) Representative field at 5 h. (E) The same field imaged at hour 6 after 1 h of exposure to control sodium chloride. (F) Representative field at 5 h. (G) The same field at h 6 after 1 h of exposure to 15 mM sodium nitrite. *P* values from one-way analysis of variance (ANOVA) followed by Sidak’s test. Three replicates per condition with at least five paired z-stacks quantified per sample at each time point.

Our goal was to develop models for studying the dispersal of P. aeruginosa biofilms in environments that recapitulate key features of the diseased human airway, including the presence of epithelial cells, apical mucus, airway surface liquid flow, and an acidic pH. Additionally, an ideal experimental model would have throughput capabilities to allow for the study of multiple conditions or strains simultaneously and be adaptable to address specific biological questions. We adapted the P. aeruginosa-epithelial coculture biofilm models described by Moreau-Marquis et al. (7) to be used for dispersal studies. Nitric oxide and NO-releasing compounds are well described triggers for biofilm dispersal in P. aeruginosa; therefore, we first tested if NO donors triggered dispersal in the coculture models (10). We cocultured the P. aeruginosa strain PAO1 on the apical surface of the CF airway epithelial cell line CFBE41o- in a perfusion chamber (schematically shown in Fig. 1A). After 6 h, distinct clusters of bacteria were seen growing on the apical surface (Fig. 1B). We imaged identical points on the cocultures at hours 5 and 6. The overall biomass increased by 5,200 ± 3,900 μm^2^ between hours 5 and 6 in control conditions (Fig. 1D and E, quantified in Fig. 1C). When 15 mM nitrite was added to the perfusate, the biomass decreased between hours 5 and 6 by 10,000 ± 5,700 μm^2^, a 50% reduction in overall biomass (Fig. 1F and G, quantified in Fig. 1C). Low mM concentrations of nitrite have been used to cause biofilm dispersal in other systems, and 15 mM sodium nitrite is below the MIC for PAO1 under aerobic conditions at pH 6.5 (13, 18). We observed a process consistent with biofilm dispersal from the surface of the airway epithelial cells.

**FIG 2:**
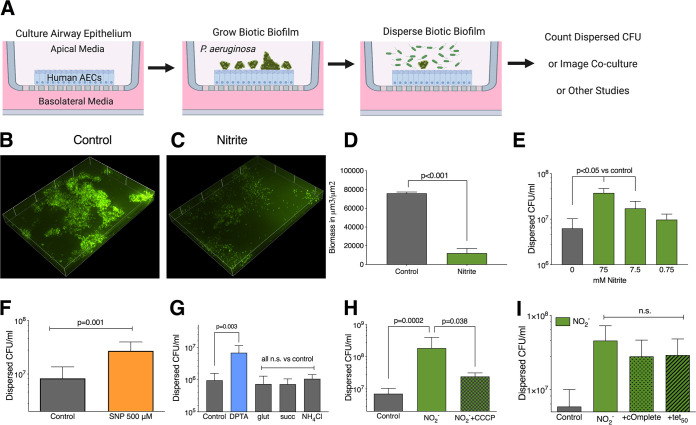
Characterization of static biotic biofilm dispersal. (A) Airway epithelial cells are cultured at the air-liquid interface, and then the apical surface is infected with P. aeruginosa. Once the coculture is mature, it is treated with a dispersal agent for 15 min, and the resulting populations can be studied further. Biofilms were grown on the apical surface of CFBE41o- airway epithelial cells for 6 h and dispersed for 15 min for all panels. Either samples were prepared for imaging or dispersed bacteria were counted by serial dilution. (B and C) Confocal scanning light microscopy images of the biotic biofilms after treatment with medium (B) or 75 mM nitrite (C). Green, PAO1-gfp. (D) Biomass quantified for at least 6 h; ×40 magnification fields from 3 samples per condition are shown; *P* value from unpaired, two-sided *t* test. (E to I) CFBE 41o- and PAO1 cocultures were treated with the indicated compounds, and the released bacteria were counted. (E) Dose response for bacterial release across 10-fold concentrations of sodium nitrite. *P* values from one-way ANOVA followed by *post hoc* Dunnett’s test. (F) Bacteria released from biofilm after treatment with 500 μM SNP versus MEM; *P* value from two-sided, unpaired *t* test. (G) Quantification of dispersed bacteria from biofilms treated with DPTA-NONOate, glutamate, succinate, or ammonium chloride. (H) Quantification of dispersed bacteria from biofilms treated with the proton ionophore CCCP and nitrite. (I) Quantification of dispersed bacteria from biofilms treated with nitrite and a protease inhibitor cocktail (cOmplete) or 50 mg/liter tetracycline. (G to I) Statistics are from one-way ANOVA followed by *post hoc* Dunnett’s test. Biologic replicates: 3 to 6 per condition tested.

We wanted to develop a second biotic biofilm assay that could accommodate a wider variety of epithelial cells and bacterial strains. We cocultured the P. aeruginosa strain PAO1 on the apical surface of polarized, well-differentiated CF airway epithelial cells (CFBE41o-) that were grown at the air-liquid interface (schematically shown in Fig. 2A). These epithelial cells produce the mucins MUC1, MUC2, MUC4, and MUC5B (37, 38). Once biofilms developed, the coculture was treated with a dispersal agent, such as sodium nitrite, for 15 min. The dispersed bacterial population was then counted, and the remaining surface-adherent population was imaged. Of note, these cocultures were previously described to have very high antimicrobial tolerance, and markers of motility are downregulated, consistent with growth as a biofilm (39, 40). Prior to treatment, a P. aeruginosa biofilm formed as an irregular mat on the epithelial surface (Fig. 2B). When the apical airway surface liquid was aspirated and replaced with 75 mM sodium nitrite, the attached biomass decreased from 75,900 ± 1,500 μm^2^/μm^3^ to 12,100 ± 5,100 μm^2^/μm^3^ (Fig. 2C). The quantitated biomass is shown in Fig. 2D (*P* < 0.05 by two-way *t* test; three replicates for each condition). Sodium nitrite, sodium nitroprusside (SNP), and NO have all been used to trigger biofilm dispersal (18). We determined the concentration range of nitrite that triggers dispersal and found a 0.8 to 1.0 log increase in the number of dispersed bacteria with 7.5 to 75 mM nitrite when the number of viable bacteria dispersed was determined by serial dilution (Fig. 2E). The use of 500 μM SNP triggered dispersal (Fig. 2F), as did the NO donor DPTA-NONOate (Fig. 2G). In minimal medium, dispersal can be triggered by changing the carbon source; however, it was unclear if changes in carbon source would trigger biotic biofilm dispersal. For these experiments, the biofilms were grown with phosphate-buffered saline (PBS) on the apical surface, rather than cell culture medium, so that any nutrients needed for growth would be derived strictly from the epithelial layer. Overall biofilm formation was unchanged compared to when minimal medium was used in the apical compartment (data not shown). Supplementing the PBS with 20 mM succinate, 20 mM glutamate, or 10 mM ammonium chloride did not trigger dispersal, while dispersal was seen with DPTA-NONOate (Fig. 2G). It is possible that the bacteria were being mechanically removed from the epithelium, rather than actively dispersing. Active dispersal classically requires flagellar motility and, thus, a proton-motive force (pmf) gradient (17). When we added the proton ionophore carbonyl cyanide m-chlorophenyl hydrazone (CCCP), which disrupts the pmf gradient, dispersal by nitrite was blocked, indicating that dispersal is an energy-dependent process (Fig. 2H). In some model systems, dispersal requires the synthesis of new proteins or protease secretion (20). We were unable to block dispersal with tetracycline or a protease inhibitor cocktail (Fig. 2I). With all this taken together, we have developed a biotic biofilm dispersal model using bacterial-epithelial coculture that demonstrates an active, energy-dependent process triggered by nitric oxide.

### Cystic fibrosis P. aeruginosa isolates have heterogenous biofilm dispersal phenotypes when grown in association with well-differentiated human primary airway epithelial cells.

**FIG 3:**
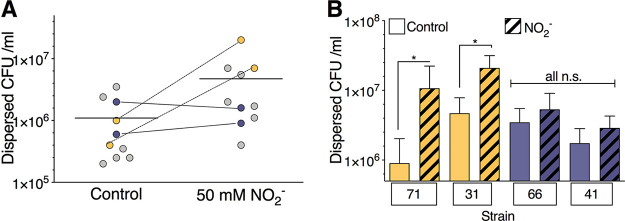
Model adaptation for primary human AECs and CF bacterial strains. (A) Biofilms were grown on the apical surface of mucus-producing primary human airway epithelial cells for 6 h. A panel of 10 CF clinical isolates was used. Biofilms were dispersed with 50 mM NO_2_^−^, and the number of dispersed bacteria were quantified by serial dilution. Several isolates did not disperse (blue), while some showed great dispersal (yellow). (B) Three to six biologic replicates of individual clinical isolates were grown as biofilms on the surface of CFBE4lo- cells and dispersed, and the dispersed bacteria were quantified. *, *P* < 0.05 by one-way ANOVA followed by Sidak’s test.

Having shown that biotic biofilms (those formed in association with airway epithelial cells) disperse in response to nitric oxide donors, we wanted to further develop the model to include primary human airway epithelial cells and CF clinical isolates. Epithelial mucus production varies with disease state and may affect bacterial mobility and behavior in the airway (41, 42). Additionally, highly evolved bacterial clinical isolates have great phenotypic and genetic diversity which is not captured through the use of laboratory strains (43). We tested a panel of 10 P. aeruginosa clinical isolates from a cystic fibrosis cohort in which individuals had chronic infection using the biotic dispersal model with primary human airway epithelial cells that produce a visible layer of mucus (44). These isolates have diverse phenotypes regarding swimming motility, lysis and sheen, abiotic biofilm formation, and mucoidy (see Table S1 in the supplemental material). There was a range of dispersal phenotypes seen, with two strains showing minimal dispersal (Fig. 3A, blue) and several strains showing >1 log of dispersal (Fig. 3A, yellow). Replicates were done of the strains at both extremes, confirming these results (Fig. 3B). Together, these data demonstrate that the biotic biofilm model allows study of dispersal in a range of bacterial clinical isolates on human primary airway epithelial cells, which could be adapted to address airway disease-specific inquiries. Moreover, heterogeneity was observed in biofilm dispersal phenotypes in the clinical isolates tested.

10.1128/mSphere.00630-20.4 TABLE S1 Phenotyping data on clinical isolates. All isolates were tested for mucoidy and lysis and sheen. The top and bottom performers in the dispersal assay were also tested for swimming and abiotic biofilm formation. Download Table S1, DOCX file, 0.01 MB. Copyright © 2020 Zemke et al. 2020 Zemke et al. This content is distributed under the terms of the Creative Commons Attribution 4.0 International license.

### The role of phosphodiesterase signaling in biofilm dispersal in a biotic biofilm.

**FIG 4:**
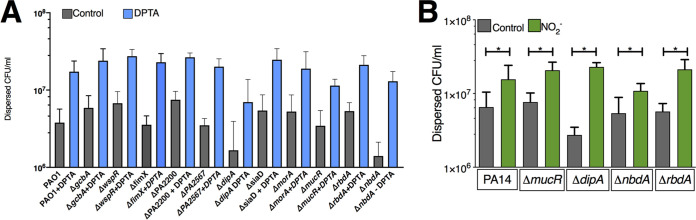
Phosphodiesterase signaling in biotic biofilm dispersal. (A) In the PAO1 background, biofilms were grown for the indicated deletion strains on CFBE41o- AECs. All deletion strains were dispersed with DPTA-NONOate. (B) In the PA14 background, biotic biofilms were grown for the indicated deletions strains. All strains were dispersed with sodium nitrite. *, *P* < 0.05 by one-way ANOVA followed by *post hoc* Sidak’s test; at least three replicates were done for each strain.

**FIG 5:**
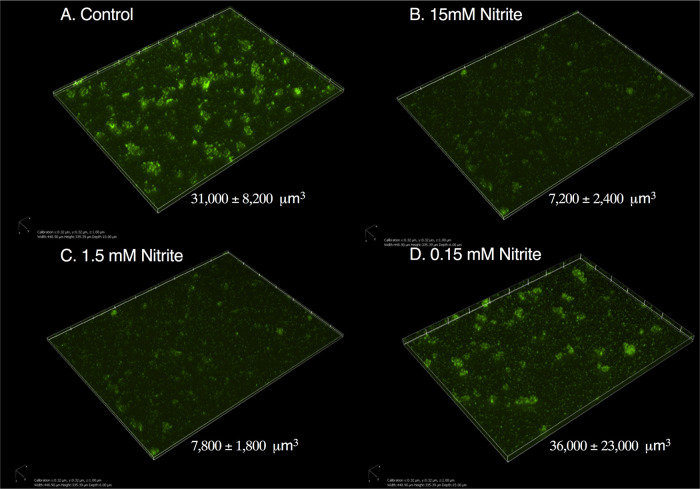
Dose response of NO-induced dispersal for biofilms grown in Fe-MEM. PAO1-gfp was grown on a glass surface in MEM supplemented with transferrin and hemoglobin and imaged after 6 h (Fe-MEM). Either sodium chloride (control) or sodium nitrite was added directly to the culture for 15 min prior to fixation. Z-stack images were collected at ×20 magnification to capture the geographic diversity of the mounts. Mean biomass ± SD is shown on each image. Three complete technical replicates were done with 6 to 10 fields imaged per replicate. (A) 15 mM sodium chloride; (B) 15 mM sodium nitrite; (C) 1.5 mM sodium nitrite; (D) 150 μM sodium nitrite.

**FIG 6:**
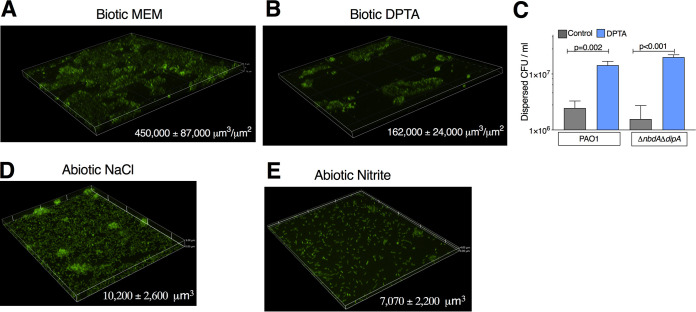
NbdA-DipA compound deletion strain dispersals. (A) Biotic biofilms of Δ*nbdA* Δ*dipA* expressing gfp were grown on CFBE41o- AECs. (B) When the biotic biofilm was treated with DPTA for 15 min, biomass decreased. Representative ×20 magnification z-stacks are shown. At least 8 fields were taken per condition. (C) Dispersed bacteria from cocultures were quantified by serial dilution; *P* values from one-way ANOVA followed by Sidak’s test. (D) Δ*nbdA* Δ*dipA* cultured on glass in Fe-MEM and rinsed once with 15 mM NaCl. (E) Abiotic culture treated with 15 mM nitrite for 15 min. Representative ×40 magnification fields. Mean biomass ± standard deviation (SD) are shown on graphs. Comparisons between conditions were significant, with *P* < 0.05 by unpaired, two-way *t* test. At least 3 biologic replicates were done for each condition.

Based on the published literature, the proteins RbdA, DipA, NbdA, and MucR are each individually required for NO-induced dispersal (19, 34, 45). Given the potential for context-dependent PDE signaling (36), we sought to determine which PDEs were required for biotic biofilm dispersal triggered by NO. We used existing transcriptomic data from our model (40) and tested dispersal of deletion strains for the most highly expressed putative PDEs/DGCs, as well as deletions of two other published PDEs (listed in Table S2) in a PAO1 background. LapDG was excluded, as it is likely an effector (26). Deletion of *dgcH* led to decreased biofilm formation, but otherwise, overall biofilm formation was not affected by deletion of the indicated genes (Fig. S1). All tested strains showed the dispersal phenotype seen in the parental strain (Fig. 4A), which was an unexpected result given what was previously published (20, 34, 46). We considered that this might be a strain effect, so we tested strains from the PA14 in-frame deletion library of possible PDEs described by Ha et al. for loss of the dispersal phenotype (29). As shown in Fig. 4B, deletion of *dipA*, *ndbA*, *mucR*, or *rbdA* did not block dispersal due to nitrite. We then screened all other strains in this library and did not find deletion of any single PDE or DGC which blocked dispersal (data not shown). Given these data, we hypothesized that *nbdA* and *dipA* might have redundant or compensatory functions in the current system. A PAO1 Δ*nbdA* Δ*dipA* strain was constructed, which had similar overall biofilm CFU as measured by serial dilution (Fig. S1). The Δ*nbdA* Δ*dipA* strain dispersed similarly to the parental strain from the surface of airway epithelial cells (Fig. 4A). To this point, all experiments were done using AEC cocultures. We then tested if strains grown in cell culture medium (minimal essential medium [MEM]) supplemented with the principal iron sources in the coculture model (transferrin and hemoglobin) behaved similarly. Note that no measurable bacterial growth occurs if the MEM is not supplemented with iron. PAO1 expressing green fluorescent protein (PAO1-gfp) was grown for 6 h on a glass coverslip with Fe-MEM (Fig. 5A). Nitrite caused dispersal of these cultures in a dose-dependent fashion between 150 μM and 15 mM (Fig. 5A to D). In this abiotic model, the biomass of the Δ*nbdA* Δ*dipA* strain decreased after 15 min of nitrite exposure, leaving behind only single attached bacteria (Fig. 6D and E). Taken together, our results indicate that biofilm dispersal in this model cannot be attributed to regulation by a single PDE/DGC and suggest functional redundancy or perhaps a cyclic-di-GMP-independent mechanism.

10.1128/mSphere.00630-20.1 FIG S1 Overall biotic biofilm formation of indicated strains as measured by CFU/ml. At least 3 replicates were done per strain. The *P* value shown is from one-way ANOVA followed by Dunnett’s test. All other comparisons to PAO1 were not statistically significant. Download FIG S1, TIF file, 0.2 MB. Copyright © 2020 Zemke et al. 2020 Zemke et al. This content is distributed under the terms of the Creative Commons Attribution 4.0 International license.

10.1128/mSphere.00630-20.5 TABLE S2 Transcriptomic data for selected possible PDE/DGCs from AEC coculture model. Download Table S2, DOCX file, 0.01 MB. Copyright © 2020 Zemke et al. 2020 Zemke et al. This content is distributed under the terms of the Creative Commons Attribution 4.0 International license.

### NO can disperse biofilms formed by P. aeruginosa with constitutively high c-di-GMP levels.

**FIG 7:**
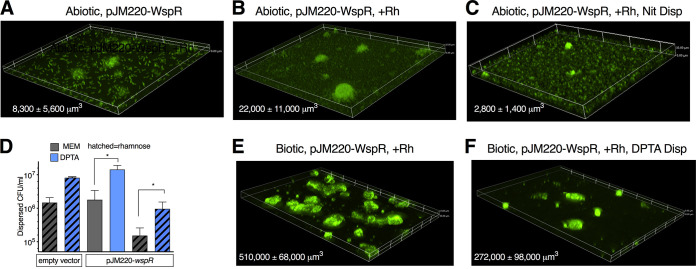
Hyper-biofilm strain dispersed with NO. (A) PAO1-pJ220-WspR grown on glass for 6 h prior to imaging; the strain expresses gfp. (B) The addition of rhamnose after the first 60 min leads to formation of distinct mounds and increased biomass. (C) 15 mM nitrite treatment of PAO1-pJM220-WspR strain with rhamnose. Changes in biomass between panels A, B, C: *P* < 0.05 by one-way ANOVA. (D) CFU counts from the indicated strains grown on AECs and dispersed with DPTA. (E) Representative image of PAO1-pJ220-WspR grown on AECs with rhamnose added after the 60-min attachment period. (F) After 15 min of exposure to DPTA, biomass drops (*P* < 0.05 by unpaired two-sided *t* test). At least three biologic replicates were done per condition.

Deletion of *dipA* caused the formation of more tightly organized areas of biofilm compared to the parental strain. The change in biofilm morphology toward tighter aggregates and increased polysaccharide production was reminiscent of that seen under high cyclic-di-GMP conditions in flow cell biofilms (47). We then asked if a hyper-biofilm-forming strain with high cyclic-di-GMP levels could be dispersed. We inserted the diguanylate cyclase *wspR* under the control of a rhamnose-inducible promoter at a single chromosomal site (pJM200-WspR) and studied this strain in our model. As expected, the strain formed increased abiotic biofilm as measured by crystal violet (Fig. S2A). In the abiotic dispersal model, the addition of rhamnose caused a 3-fold increase in biomass at 6 h (Fig. 7A versus Fig. 7B). However, treatment with nitrite for 15 min still led to a 90% reduction in biomass (Fig. 7C). Using confocal microscopy, we saw that nitrite treatment led to a more disordered appearance of the attached cells between the biofilms, as well as a decrease in the area covered with bacteria (Fig. S2B to F). We then tested this strain in our biotic biofilm model. We allowed PAO1-pJM220-WspR to attach for 1 h prior to adding rhamnose for the remaining 5 h. The resulting biofilms formed areas of highly organized clumps not present in empty vector control biofilms (Fig. 7E). In the dispersal assay, 1 log fewer bacteria were removed within 15 min by medium change alone in the presence of rhamnose (Fig. 7D), yet the NO donor DPTA-NONOate still led to an 8-fold increase in dispersed bacteria and a 50% decrease in biomass (Fig. 7D and F). From these data, we conclude that altering cyclic-di-GMP signaling results in morphological change in biotic biofilms, but the NO-mediated dispersal response is preserved. These results provide additional evidence of c-di-GMP-independent mechanisms regulating biotic biofilm dispersal.

10.1128/mSphere.00630-20.2 FIG S2 (A) Crystal violet biofilm assay results for indicated strains and rhamnose. *P* < 0.05 by one-way ANOVA. Three replicates were done. (B) Quantification of the percentage area covered in panels C to F. Statistics from one-way ANOVA followed by Sidak’s test. (C to F) Representative ×40 magnification fields for the indicated conditions and strains. Download FIG S2, TIF file, 1.6 MB. Copyright © 2020 Zemke et al. 2020 Zemke et al. This content is distributed under the terms of the Creative Commons Attribution 4.0 International license.

### Dispersal by NO has differential effects on antibiotic efficacy.

**FIG 8:**
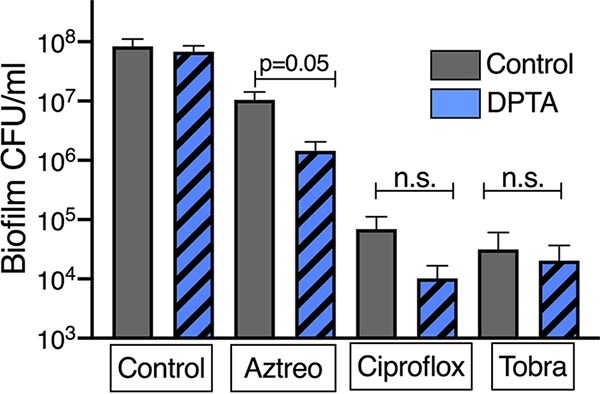
Interaction between NO-triggered dispersal and antibiotics. Biotic biofilms were grown for 6 h on CFBE41o- cells. Cocultures were dispersed for 15 min with DPTA-NONOate, and then antibiotics were added for an additional 20 h. Adherent bacteria were counted by serial dilution and analyzed with one-way ANOVA followed by Dunnett’s test, with at least three replicates per condition.

Biofilm lifestyle confers high antimicrobial tolerance, and ongoing translational work focuses on combining a biofilm dispersal agent (generally an NO donor) with antibiotics (48). We tested the hypothesis that pretreating the biotic biofilms with the NO would increase antibiotic killing by increasing the dispersed, and thus more antibiotic-sensitive, bacterial population. Biotic biofilms were treated for 15 min with NO donor DPTA-NONOate and then with the commonly used antipseudomonal antibiotics ciprofloxacin, tobramycin, or aztreonam for 20 h. DPTA-NONOate combined with aztreonam decreased by 1 log the viable adherent bacteria (Fig. 8). Pretreatment with DPTA-NONOate had no effect on biofilm killing by ciprofloxacin or tobramycin after a 20-h incubation period (Fig. 8). We (and others) previously reported induction of tolerance to aminoglycosides with high concentrations of NO (49, 50). At 90 min of tobramycin exposure, there was a striking tolerance in planktonic population (Fig. S3), but by 18 h, the apical supernatant had colony counts below the limit of detection. Considering these results taken together, we found that combining NO donors with existing antimicrobials yields various degrees of killing, with the promising observation of that the combination of NO with aztreonam increases biofilm clearance.

10.1128/mSphere.00630-20.3 FIG S3 PAO1 biofilms were grown for 6 h and treated with DPTA for 15 minutes, and then tobramycin was added for 90 min. The remaining attached and dispersed bacteria were counted by serial dilution. For the control well (not exposed to DPTA), the bacterial count in the apical supernatant was below the limit of detection. Download FIG S3, TIF file, 0.1 MB. Copyright © 2020 Zemke et al. 2020 Zemke et al. This content is distributed under the terms of the Creative Commons Attribution 4.0 International license.

## DISCUSSION

We developed biofilm dispersal models that simulate aspects of the airway environment. Using these three models, nitric oxide uniformly stimulates biofilm dispersal. However, importantly, we found that the roles of specific phosphodiesterases in mediating biofilm dispersal differ between biofilms grown in these nutritional environments and what has been published from flow cells using minimal medium. We adapted the static AEC coculture model to study bacterial clinical isolates and use primary human airway epithelial cells. Finally, we used the model to extend our understanding of the effects of combining a dispersal agent and antibiotic tolerance. NO improved clearance by aztreonam, while with tobramycin we saw a transient induction of tolerance that resolved when the incubations were extended longer. These data provide proof of concept for the importance of studying bacterial community processes such as biofilm dispersal in environments that more closely resemble chronic disease states.

Nitric oxide has been extensively studied as a P. aeruginosa biofilm dispersal agent *in vitro*, and more recently, NO dispersal has been shown in expectorated sputum samples from individuals exposed to NO (10, 16, 34, 51). We saw dispersal with DPTA-NONOate, which decomposes to two NO molecules, as well as acidified sodium nitrite and sodium nitroprusside. Previous work had used 15 mM NaNO_2_, and we saw dispersal at 15 mM, as well as the 50 to 75 mM concentrations we calculate may be generated with nebulization of nitrite. Dispersal was seen within 15 min, a time period shorter than the generation time of P. aeruginosa, and the result was experimentally robust across models. We did not see dispersal when other carbon sources were added to the medium, which suggests that nutrient-induced dispersal is dependent on the abundance and variety of available nutrition sources. We are able to block dispersal with the addition of a proton ionophore, CCCP (carbonyl cyanide *m*-chlorophenylhydrazone), which disrupts the proton gradient needed for flagellar motility (as well as other processes). CCCP was previously shown to block dispersal due to glucose starvation, and it reinforces the conclusion that dispersal seen from AECs is an active process rather than just due to mechanical disruption (52).

The switch between motile and attached lifestyles is regulated by the second messenger cyclic-di-GMP at multiple levels. NO leads to increased phosphodiesterase activity with a corresponding decrease in cyclic-di-GMP levels (34, 51). In flow cells, deletion of the phosphodiesterase DipA, NbdA, RbdA, or MucR or the chemosensory protein BdlA in isolation is sufficient to block NO-induced dispersal (20, 34, 51). Both NbdA and DipA are transcriptionally upregulated during flow cell dispersal, and in flow cells, dispersal was blocked by tetracycline, suggesting that ongoing protein synthesis was required (20). In contrast, we did not find that any single PDE was required for NO dispersal in either PAO1 or PA14 in these models. The nutritional environment may be a key contributing factor. For example, the effects of deletion of *dipA* on swimming and swarming motility varied across a panel of minimal and rich media tested (36). Additionally, there may be functional redundancy or compensatory activity at the PDE/DGC level. For example, deletion of PA3177 results in compensatory upregulation of other DGC genes in the setting of hypochlorite stress (11). Finally, an abiotic biofilm dispersal model of pyruvate depletion was recently published that appears to be independent of the previously identified phosphodiesterases, despite being studied within a well-described abiotic system (53), further supporting that additional signaling mechanisms may be at play.

While we were unable to block dispersal through deletion of any single PDE, we do find evidence that cyclic-di-GMP affects community morphology and dispersal in the biotic biofilm model. Deletion of *dipA* led to more tightly clumped aggregates, as did overexpression of *wspR*. We also acknowledge that overexpression of WspR causes additional phenotypes, including increased aggregation and matrix production. Despite this, NO was still able to trigger dispersal in the Δ*dipA* and pJM220-*wspR* strains, although the number of bacteria released with a simple medium change was lower, possibly reflecting increased physical durability of the biofilms in the hyper-biofilm strains. In other models, overexpression of the Escherichia coli PDE *yhjH* triggers abiotic dispersal; however, endogenously expressed PDEs have additional layers of spatial organization or other levels of regulation not captured in heterologous expression studies (54). Closely related cyclic-di-GMP proteins also appear to have functional outputs that are not solely predicted by their role in regulating basal cyclic-di-GMP levels (45). Alternatively, there may be other as yet unidentified signaling pathways involved.

The airway epithelium is highly decorated with a complex network of mucins, both tethered and secreted. During chronic airway disease, abundant mucus can fill the lumen, and disease states such as asthma, chronic obstructive pulmonary disease, and cystic fibrosis may have disordered mucus secretion and mucus of abnormal viscoelastic properties (reviewed in reference 55). The CFBE41o- cell line used for most experiments expresses surface-attached mucins but does not differentiate into goblet cells under the conditions used in the current study. Environmental viscosity may participate in community formation; thus, we wanted to examine biofilm dispersal in the presence of secreted mucus (41). We observed dispersal of clinical isolates grown on primary human airway epithelial cells that produced macroscopic mucus in culture, although results varied between individual bacterial clinical isolates. That diversity in dispersal response was seen among a panel of CF clinical isolates is not surprising given the enormous phenotypic diversity seen even within a single chronically infected lung (43). Variability in endogenous dispersal was previously seen in five clinical isolates, and there is some limited evidence that *in vivo* dispersal occurs following NO inhalation in cystic fibrosis (16, 22). It remains to be determined what portion of the bacterial community is susceptible to dispersal agents *in vivo*, either within an individual patient or between patients.

The combination of antibiotics and dispersal cues has been proposed for some time as a treatment for biofilm-based infections (16, 48, 56). Biofilm antimicrobial tolerance is complex, with roles for cyclic-di-GMP-regulated drug efflux, sequestration by matrix, and low metabolic rate all implicated as contributing to biofilm-mediated tolerance (33, 57, 58). Many studies pairing dispersal and antibiotics have used NO triggers, although these studies have varied in regard to the specific source of nitrosative stress, antibiotic concentrations, treatment duration, medium, measurement of bacterial viability, and the presence of bulk flow (16, 48). We saw no interaction between NO and ciprofloxacin or tobramycin at 20 h. There was an additive effect between NO and aztreonam and striking tolerance to tobramycin among the dispersed population in the presence of NO at 6 h, which resolved by 20 h. At concentrations of NO high enough to inhibit bacterial respiration, antagonism between nitrosative stress and the aminoglycosides has been well documented in multiple species (49, 50, 59, 60). Our data suggest that the choice of particular antibiotic combined with a dispersal agent may be important if nitric oxide is used. Other dispersal approaches, such as matrix-degrading enzymes, cis-2-decanoic acid, pyruvate depletion, or directly influencing cyclic-di-GMP, may have fewer potential interactions with antibiotics (61–63) and could be examined in future studies.

The three models described in this study each have unique strengths and limitations. Coculture of P. aeruginosa on airway cells in perfusion chambers allows for studies of flow-dependent behaviors and visualization in real time; however, only 2 to 4 chambers can be feasibly studied by a single investigator per day, and the studies require a live-cell microscopy system. Coculture of P. aeruginosa on AECs grown on Transwells provides the opportunity to study the effects of various epithelial pathologies, allows the use of a wide variety of clinical isolates, may include both secreted and tethered mucins, and is amenable to transfection experiments (64). A limitation is the large amount of human nucleic acids present, which complicates, but does not exclude, bacterial transcriptomic studies. Growth of biofilms on glass in cell culture medium required the addition of iron, which is likely derived from the epithelial cells in our biotic biofilm models. However, fundamental behaviors during PDE manipulations replicated well in this model, and it may be more amenable to study designs requiring serial passaging such as *in vivo* evolution or Tn-Seq experiments. We would also reinforce that the fundamental behavior of bacterial community dispersal by nitric oxide replicated uniformly across all the systems we studied, which is very encouraging for its clinical application. In conclusion, P. aeruginosa communities cultured either with human airway epithelial cells or in cell culture medium supplemented with transferrin and hemoglobin are dispersed in response to nitric oxide donors. The novel epithelial-bacterial dispersal model described here advances our understanding of bacterial biofilm dispersal in the environment of the host. Looking forward, the model provides a setting that better replicates the airways for elucidating dispersal signaling in more complex environments, identifying other dispersal triggers, or studying the interactions between dispersal triggers and antibiotics as new therapeutics to treat chronic bacterial infections.

## MATERIALS AND METHODS

### Reagents.

Reagents were purchased from Sigma Chemical unless listed as follows: DPTA-NONOate (Caymen Chemicals), tobramycin (Fresenius Kabi), aztreonam (Alfa Aesar), Hoechst 33342 (Invitrogen), cOmplete protease inhibitor (Roche).

### Strains and growth conditions.

Overnight cultures were routinely grown in lysogeny broth (LB; Sigma) at 37°C on a roller drum. Deletion strains were made in the P. aeruginosa PAO1 background using standard molecular cloning techniques. In-frame deletion strains were constructed using homologous recombination in the protocol described in reference 65 with pMQ30 as the allelic replacement vector (66). Primers and strains are listed in Table S3. Briefly, the upstream and downstream regions adjacent to the gene to be deleted were amplified using PCR and ligated into pMQ30. Sequence-confirmed allelic replacement plasmids were mated into PAO1, and gentamicin/sucrose were used for selection/counterselection. To confirm final strain identity, whole-genome sequencing was done on a NextSeq 500 system (Illumina) using 2 × 150-bp libraries at University of Pittsburgh’s Microbial Genome Sequencing Center. Breseq version 0.28.1 was used for variant calling with alignment to the PAO1 genome (67). No additional mutations were found in the strains used. PAO1-pJM220-WspR was created by inserting the open reading frame of *wspR* into pJM220 using standard cloning techniques. The sequence-confirmed plasmid was mated into the recipient PAO1 strain with pTNS3 and pRK2013. Insertion of the rhamnose-inducible *wspR* cassette at the *attTn7* site was confirmed by PCR (68).

10.1128/mSphere.00630-20.6 TABLE S3 Bacterial strains and primers used. Download Table S3, DOC file, 0.09 MB. Copyright © 2020 Zemke et al. 2020 Zemke et al. This content is distributed under the terms of the Creative Commons Attribution 4.0 International license.

### Human cell culture and biotic biofilm dispersal model.

The biotic biofilm dispersal protocol was modified from Moreau-Marquis et al. (7). Unless otherwise indicated, the CFBE41o- immortalized human bronchial epithelial cell line homozygous for F508del-CFTR was used (69).

For the flow model, cocultures were grown as described in reference 64 with the following modifications. Confluent CFBE41o- epithelial cells were cultured on sterile glass coverslips for 7 to 10 days prior to the experiment. Coverslips were rinsed to remove antibiotics, and a Hoechst nuclear counterstain was applied. Perfusion chambers were inoculated with PAO1 expressing gfp as previously described (7), and the bacteria were allowed to attach for 60 min with the perfusion pump stopped. After a 1-h attachment period, the perfusion pump was restarted for the rest of the experiment. Live, wide-field microscopy was used to collect images. At least five z-stack images were taken after 5 h and again after 6 h in the same locations at ×40 magnification. Z-stacks were 20 to 25 μm in depth. At hour 5, either 15 mM sodium chloride or 15 mM sodium nitrite was added to the perfusate. The transit time to the chamber was 15 to 20 min, resulting in a 40-min exposure. Images were quantified using Nikon Elements software. Absolute biomass at hours 5 and 6 (± nitrite) were compared using one-way analysis of variance (ANOVA).

CFBE41o- airway epithelial cells (AECs) were seeded at confluence on 12-mm Transwell permeable membrane supports (Corning) and grown at the air-liquid interface for 5 to 9 days prior to experimentation in minimal essential medium (MEM) supplemented with fetal bovine serum, penicillin, streptomycin, and plasmocin. The morning of the experiment, overnight cultures were rinsed and resuspended in MEM, AECs were rinsed twice with MEM to remove residual antibiotics, and the basolateral compartment was replaced with MEM. Rinsed bacteria were diluted to a multiplicity of infection of 1:25 in 500 μl MEM and allowed to attach to the apical epithelial surface for 60 min at 37°C in a cell culture incubator with 5% CO_2_ atmosphere. The apical compartment was aspirated and replaced with 500 μl fresh MEM + 0.1% arginine, and the coculture was incubated for an additional 5 h. For overall biofilm formation, the apical compartment was aspirated and rinsed once gently with MEM, and adherent bacteria were removed with 0.1% Triton X-100 and then vortexed for 3 min. Viable bacteria were counted by serial dilution. The lower limit of detection was 10^2^ CFU/ml.

For biotic biofilm dispersal assays, after the 5-h incubation, the apical compartment was gently aspirated and replaced with prewarmed medium containing the dispersal agent. After 15 min, the apical compartment was removed, added to an equal volume of 0.1% Triton X-100 in MEM, and vortexed for 3 min, and viable bacteria were counted using serial dilution. In some experiments, the remaining adherent bacteria were also counted to determine overall biofilm formation. For experiments with DPTA-NONOate, the DPTA-NONOate was allowed to equilibrate in pH 7.2 cell culture medium at 37°C for at least 1 h prior to use. Sodium nitrite was used at 15 to 75 mM with pH 6.5 medium (to mimic the pH of the CF airway). Equimolar sodium chloride was used as a tonicity control in sodium nitrite experiments.

To test the combination of DPTA-NONOate and antibiotics, the biofilm dispersal assay was extended. After the DPTA-NONOate had incubated for 15 min, the antibiotics were added directly to the wells at the following final concentrations: 5 μg/ml ciprofloxacin, 1,000 μg/ml tobramycin, and 500 μg/ml aztreonam. After further incubation (90 min to 20 h), viable bacteria in the apical compartment and adherent bacteria were counted as described above.

For dispersal experiments done using primary human airway epithelial cells (HBE), well-differentiated HBEs grown at the air-liquid interface were obtained from the Airway Cell Core at University of Pittsburgh, and the dispersal assay was otherwise conducted without protocol modifications. Studies were covered under University of Pittsburgh Institutional Review Board approval number IRB970946.

### Imaging.

Biotic biofilms were grown and dispersed as described above using strains carrying a constitutive expressing green fluorescent protein plasmid. Cocultures were stained with Hoechst 33342 dye to visualize the epithelial nuclei, fixed with 4% paraformaldehyde overnight at 4°C, and mounted on slides with Floromount (Invitrogen). Confocal laser scanning microscopy was used to obtain Fig. 2B and C. Biotic biofilms in subsequent figures were imaged on a wide-field Nikon microscope at ×20 magnification. Images were deconvoluted using identical settings across all images. Volumetric projections were rendered using Nikon Elements software. For quantification, at least 6 random z-stack fields were taken with a Nikon Ti inverted microscope. Z-stacks had a threshold applied and the volume determined using Nikon Elements version 4.30.02 software. For percentage area covered (Fig. S2), ×40 magnification images were taken at the focal plane of the monolayer bacteria, and a threshold was applied to the image. At least six images were taken per replicate.

### Abiotic model.

The morning of the experiment, overnight cultures were rinsed and resuspended in MEM and diluted 1:4 in MEM for a total volume of 500 μl. Iron-supplemented medium containing 62.5 μM human holotransferrin and 31.25 μM human hemoglobin in MEM plus l-Glu plus l-Arg was used as an iron source for bacterial biofilm growth (Fe-MEM). The bacteria were diluted again (7:250) in Fe-MEM and added to the center of a MatTek glass-bottom dish. If rhamnose induction was used, the bacteria were incubated for 1 h at 37°C with 5% CO_2_ on the MatTek dish before addition of 1% rhamnose and then incubated further for 5 h. After incubation, sodium nitrite was added at 0.15 to 15 mM to the treatment dish to induce dispersal and incubated for an additional 15 min. After dispersal, the MatTek dishes were fixed with 2.5% glutaraldehyde in PBS at 4°C under foil overnight. The next day, the fixative was removed, and cultures were mounted in ProlongGold. Z-stack images were obtained using a Nikon Ti inverted microscope, and quantification was performed using Nikon Elements version 4.30.02 software. All strains used expressed gfp.

### Crystal violet assay.

Abiotic biofilms were grown on plastic microtiter plates as described in reference 70. Overnight cultures were normalized to the optical density (OD) of the least dense strain for the day and diluted 1:33 in LB broth, and 100 μl of the dilution was placed in a Costar 2797 polyvinyl chloride plate. The plate was incubated for 24 h at 37°C without shaking and then stained with crystal violet as described in reference 71.

### Clinical isolate phenotyping.

Swimming assays were performed using 0.3% LB agar plates incubated at 37°C. For mucoidy determinations, strains were grown at 37°C overnight and then for at least 48 h at room temperature. For lysis and sheen determinations, strains were grown for at least 48 h prior to determination.

### Statistics.

At least three replicates were done of all experiments, and typically 5 to 10 replicates were completed. Statistical analysis was done using Prism version 8.0 software (GraphPad, San Diego, CA). Data are displayed as the mean ± the standard deviation. CFU counts were log transformed, and then either a *t* test or one-way ANOVA was done, depending on the experimental design.
