# Supplementary material for: Dispersal of Epithelium-Associated Pseudomonas aeruginosa Biofilms
Source: mSphere. 2020 Jul 15;5(4):e00630-20. doi: 10.1128/mSphere.00630-20 (PMC7364222; doi:10.1128/mSphere.00630-20)
Supplement: TABLE S1 [file mSphere.00630-20-st001.docx]

**Supplemental Table 1**

Phenotyping Data on Clinical Isolates

| Isolate | Mucoid | Lysis and Sheen | Swimming diameter mm | Abiotic Biofilm Formation (O.D. _600_) mean (s.d) |
| --- | --- | --- | --- | --- |
| 71-2 | No | No | 29 | 0.23 ± 0.08 |
| 31-2 | No | No | 31 | 0.096 ± 0.019 |
| 66-3 | Yes | No | 7.5 | 0.20 ± 0.09 |
| 41-2 | No | Yes | 24 | 0.22 ± 0.04 |
| 36-3 | No | No |  |  |
| 62-3 | No | No |  |  |
| 74-2 | Yes | Yes |  |  |
| 47-3 | Yes | Yes |  |  |
| 33-2 | No | Yes |  |  |
| 60-3 | No | No |  |  |
