# Supplementary figures and images for: Dispersal of Epithelium-Associated Pseudomonas aeruginosa Biofilms
Source: mSphere. 2020 Jul 15;5(4):e00630-20. doi: 10.1128/mSphere.00630-20 (PMC7364222; doi:10.1128/mSphere.00630-20)

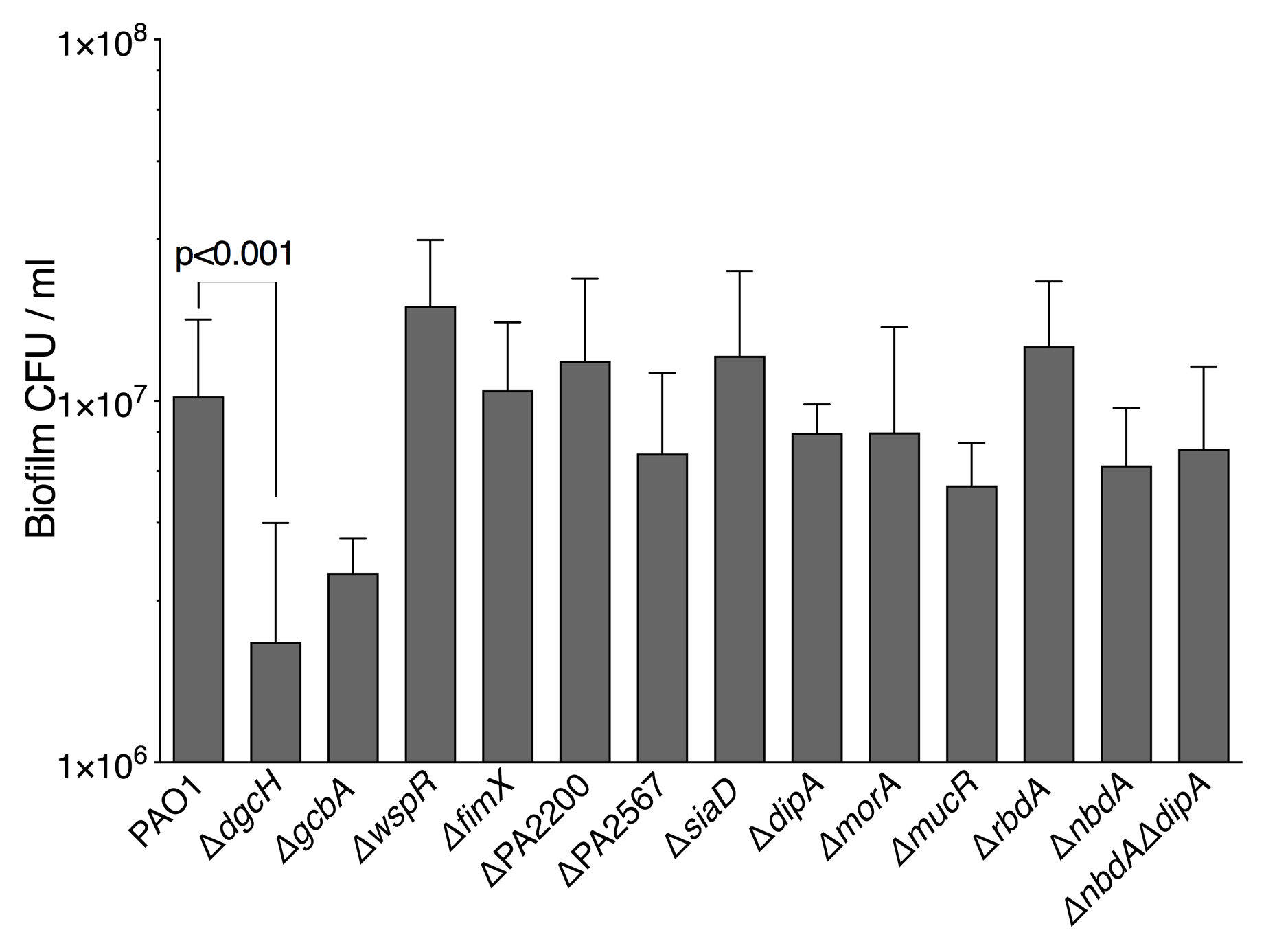

Supplement: FIG S1 [file mSphere.00630-20-sf001.tif]

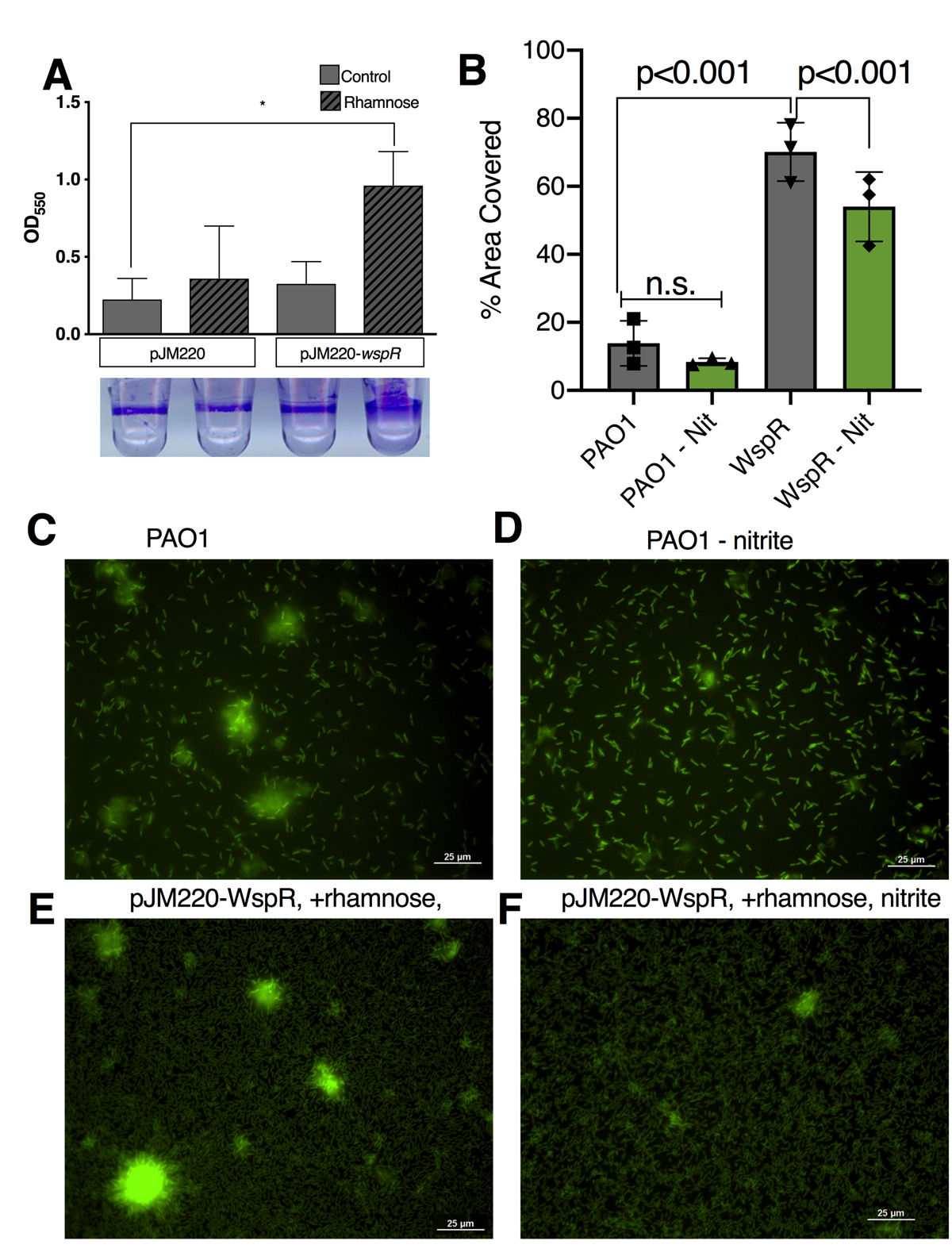

Supplement: FIG S2 [file mSphere.00630-20-sf002.tif]

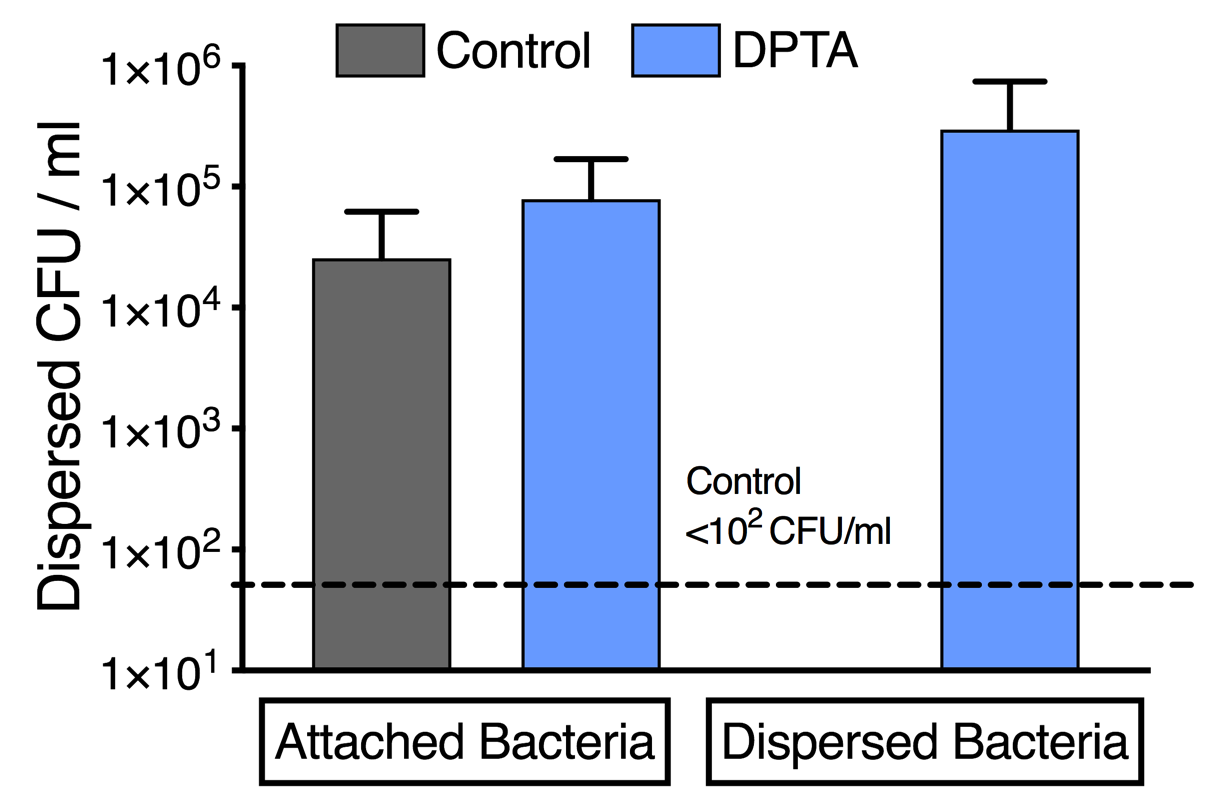

Supplement: FIG S3 [file mSphere.00630-20-sf003.tif]
