# Supplementary material for: Dispersal of Epithelium-Associated Pseudomonas aeruginosa Biofilms
Source: mSphere. 2020 Jul 15;5(4):e00630-20. doi: 10.1128/mSphere.00630-20 (PMC7364222; doi:10.1128/mSphere.00630-20)
Supplement: TABLE S2 [file mSphere.00630-20-st002.docx]

Supplemental Table 2: Transcriptomic data for selected possible PDE/DGC’s from AEC coculture model.

| **Gene ID** | **Gene Name** | **RPMK** |
| --- | --- | --- |
| PA4843 | *gcbA* | 448 |
| PA3702 | *wspR* | 288 |
| PA4959 | *fimX* | 217 |
| PA2200 | *--* | 202 |
| PA2567 | *--* | 122 |
| PA5487 | *dgcH* | 112 |
| *PA1433* | *lapG* | *98* |
| PA5017 | *dipA* | 93 |
| PA0169 | *siaD* | 84 |
| PA4601 | *morA* | 75 |
| PA1727 | *mucR* | 60 |
| PA0861 | *rbdA* | 49 |
| PA3311 | *nbdA* | 4 |
