# Supplementary material for: Dispersal of Epithelium-Associated Pseudomonas aeruginosa Biofilms
Source: mSphere. 2020 Jul 15;5(4):e00630-20. doi: 10.1128/mSphere.00630-20 (PMC7364222; doi:10.1128/mSphere.00630-20)
Supplement: TABLE S3 [file mSphere.00630-20-st003.doc]

Supplemental Table 3: Bacterial Strains and Primers Used

Bacterial strains used:

| ***P. aeruginosa* Strain** | **Description** | **Source** |
| --- | --- | --- |
| PAO1 | WT | G. O’Toole |
| PA14 | WT | G. O’Toole |
| PAO1-gfp | PAO1 with a constitutive gfp plasmid | G. O’Toole |
| Phosphodiesterase and diguanylyl cyclase library | | Described in (1) |
| Late cystic fibrosis clinical isolates | | Described in (2) |
| Δ*nbdA* | In-frame deletion of *nbdA* | This paper |
| Δ*dipA* | In-frame deletion of *dipA* | This paper |
| Δ*gcbA* | In-frame deletion of *gcbA* | This paper |
| Δ*wspR* | In-frame deletion of *wspR* | This paper |
| Δ*fimX* | In-frame deletion of *fimX* | This paper |
| Δ*PA2200* | In-frame deletion of *PA2200* | This paper |
| Δ*PA2567* | In-frame deletion of *PA2567* | This paper |
| Δ*dgcH* | In-frame deletion of *dgcH* | This paper |
| Δ*morA* | In-frame deletion of *morA* | This paper |
| Δ*mucR* | In-frame deletion of *mucR* | This paper |
| Δ*rbdA* | In-frame deletion of *rbdA* | This paper |
| Δ*siaD* | In-frame deletion of *siaD* | This paper |
| Δ*nbdA*ΔdipA | In-frame deletion of *nbdA* and *dipA* | This paper |
| PAO1-pJM220 | PAO1 *attTn7::*pJM220 | This paper |
| PAO1-pJM220-WspR | PAO1 *attTn7::*pJM220-WspR | This paper |
| **Plasmid** | **Description** | **Source** |
| pMQ30 |  | (3) |
| pTNS3 |  | (4) |
| pRK2013 |  | (5) |
| pJM220 | pUC18T-miniTn7T-gm-rhaSR-PrhaBAD | (6) |
| pJM220-WspR | pJM220 with coding sequence of WspR | This study |

| **Primer** | **Sequence** |
| --- | --- |
| DipAUpF | cggaattcgctgtcgctgtcctacgaccatc |
| DipAUpR | gggcgatcagtgcagggtggcgaggctgattccaggtactg |
| DipADownF | cagtacctggaatcagcctcgccaccctgcactgatcgccc |
| DipADownR | gcaagaattcgctttcgccagttcggc |
| GcbAUpF | ggggacaagtttgtacaaaaaagcaggctacaaccagcatcgccaccag |
| GcbAUpR | cggttgatgacaaaaggccagacgcgcttctttcgtggt |
| GcbADownF | accacgaaagaagcgcgtctggccttttgtcatcaaccg |
| GcbADownR | ggggaccactttgtacaagaaagctgggtaccaattcgcgctcgacgt |
| GcbAExtF | gcatgttgtccatccagatgcc |
| GcbAExtR | gcaactcgtccggatagcgtt |
| WspRUpF | ggggacaagtttgtacaaaaaagcaggctaccctggatgtccaggtaggcg |
| WspRUpR | gtcccggagagaaacatgccctgatggaacagccggt |
| WspRDownF | accggctgttccatcagggcatgtttctctccgggac |
| WspRDownR | ggggaccactttgtacaagaaagctgggtagcaaggtccaggtggtgc |
| WspRExtF | atctcggtgcgcagccagc |
| WspRExtR | gcggaaagtccttgcgcgat |
| FimXUpF | ggggacaagtttgtacaaaaaagcaggctacggactacccgacctacgcc |
| FimXUpR | ggcgcccgttcttcattcgggaaagggctcagtccgcg |
| FimXDownF | cgcggactgagccctttcccgaatgaagaacgggcgcc |
| FimXDownR | ggggaccactttgtacaagaaagctgggtacgccgaactcaagcgcct |
| FimXExtF | ccttcgatgcacagaaggtcac |
| FimXExtR | cggtgaactggacttccgc |
| PA2200UpF | ggggacaagtttgtacaaaaaagcaggctacatcgagcaagtcaaggcg |
| PA2200UpR | cggaaggtccgttctagccgttccacgctcggacat |
| PA2200DownF | atgtccgagcgtggaacggctagaacggaccttccgc |
| PA2200DownR | ggggaccactttgtacaagaaagctgggtatgcagcgcgaacggatact |
| PA2200ExtF | gccggcaagctgaacat |
| PA2200ExtR | aagaacgcccgcaactg |
| PA2567UpF | ggggacaagtttgtacaaaaaagcaggctacgcctgagaagacccgcc |
| PA2567UpR | gtgcctgcgttggctaacggctggactcctgggcta |
| PA2567DownF | tagcccaggagtccagccgttagccaacgcaggcac |
| PA2567DownR | ggggaccactttgtacaagaaagctgggtagcgattacagttgcgatggc |
| PA2567ExtF | ggtagagccggacgacca |
| PA2567ExtR | gctctcttctccattgccgc |
| DgcHUpF | ggggacaagtttgtacaaaaaagcaggctacccacagcggaccgtcgaag |
| DgcHUpR | ggagtccgccatgagtcggcctgaagggcgaccag |
| DgcHDownF | ctggtcgcccttcaggccgactcatggcggactcc |
| DgcHDownR | ggggaccactttgtacaagaaagctgggtagccttctcagcttcaacatcc |
| DgcHExtF | cgcaggttgtccaacagcatat |
| DgcHExtR | cgagaaagagcgcgcagc |
| MorAUpF | ggggacaagtttgtacaaaaaagcaggctaccgtaagcaaggccaccctg |
| MorAUpR | ctcgttgaacatgaacagcacgcgacggggttagcgaggg |
| MorADownF | ccctcgctaaccccgtcgcgtgctgttcatgttcaacgag |
| MorADownR | ggggaccactttgtacaagaaagctgggtacgttgtacagcttgccggag |
| MorAExtF | cgatccttcctattgcacgca |
| MorAExtR | ggttcgggtagacgccag |
| MucRUpF | ggggacaagtttgtacaaaaaagcaggctaccgagaagctcatgtcgagcc |
| MucRUpR | cgttcggacgctttcatgccgagcagttgctcgccag |
| MucRDownF | ctggcgagcaactgctcggcatgaaagcgtccgaacg |
| MucRDownR | ggggaccactttgtacaagaaagctgggtacccagtctgaaggaaagcc |
| MucRExtF | ccgttgatggtgttcagcgag |
| MucRExtR | gcaggtcgtcgttgacgc |
| RbdAUpF | cgtaGAATTCtggcgggctttggagat |
| RbdAUpR | cacctaccggaggttctgtctccatctaccattcaaactggcg |
| RbdADownF | cagtttgaatggtagatggagacagaacctccggtaggtg |
| RbdADownR | cgttGAATTCtcgatggcgtagttggtgg |
| RbdAExtF | caactgctactcggcctctaca |
| RbdAExtR | tactgggcgtggagcttga |
| NbdAUpF | atggagccggaacaagctcagctggttcactcgacggcggtccg |
| NbdAUpR | cggaccgccgtcgagtgaaccagctgagcttgttccggctccat |
| NbdADownF | ccgaattccggtggcgttggtctcgaagac |
| NbdADownR | cggaattcgctggcggtgtggaaccgttc |
| NbdAExtF | cgtctatcggctcgtcc |
| NbdAExtR | cgtgcccattcgctgc |
| siaDUpF | ggggacaagtttgtacaaaaaagcaggctacctcaagggctacggcgaaca |
| siaDUpR | ggctggacgcctgaggagcagttgctccagcgattgctg |
| siaDDownF | cagcaatcgctggagcaactgctcctcaggcgtccagcc |
| siadDownR | ggggaccactttgtacaagaaagctgggtaccgtgcacctgatcttcct |
| siaDExtF | agctacacccgccaacgc |
| siaDExtR | atttcaaccaggactaccgcagc |
| NbdDownF2 | atggagccggaacaagctcagctggttcactcgacggcggtccg |
| NbdUpR2 | cggaccgccgtcgagtgaaccagctgagcttgttccggctccat |
| NbdUpF2 | ccgaattccggtggcgttggtctcgaagac |
| NbdDownR2 | cggaattcgctggcggtgtggaaccgttc |
| WspRFwd | tcacactagtcccggagagaaacatgcacaa |
| WspRRev | gcacaagctttatactgcacttgcgcccc |

**Works Cited**

1. Ha D-G, Richman ME, O’Toole GA. 2014. Deletion mutant library for investigation of functional outputs of cyclic diguanylate metabolism in *Pseudomonas aeruginosa* PA14. Appl Environ Microbiol 80:3384 –3393.<https://doi.org/10.1128/AEM.00299-14>.
2. Smith EE, Buckley DG, Wu Z, Saenphimmachak C, Hoffman LR, D’Argenio DA, Miller SI, Ramsey BW, Speert DP, Moskowitz SM, Burns JL, Kaul R, Olson MV. 2006. Genetic adaptation by *Pseudomonas aeruginosa* to the airways of cystic fibrosis patients. Proc Natl Acad Sci U S A 103:8487– 8492. https://doi.org/10.1073/pnas.0602138103.
3. Shanks RMQ, Caiazza NC, Hinsa SM, Toutain CM, O’Toole GA. 2006. Saccharomyces cerevisiae-based molecular tool kit for manipulation of genes from Gram-negative bacteria. Appl Environ Microbiol 72: 5027–5036. <https://doi.org/10.1128/AEM.00682-06>.
4. Choi KH, Mima T, Casart Y, Rholl D, Kumar A, Beacham IR, Schweizer HP. 2008. **Genetic tools for select-agent-compliant manipulation of Burkholderia pseudomallei.** Appl Environ Microbiol. 2008 Feb;74(4):1064-75. https://doi.org/[10.1128/AEM.02430-07](https://doi.org/10.1128/aem.02430-07)
5. Figurski DH, Helinski DR. 1979. Replication of an origin-containing derivative of plasmid RK2 dependent on a plasmid function provided in trans. Proc. Natl. Acad. Sci. USA 76: 1648-1652, 1979. <https://doi.org/10.1073/pnas.76.4.1648>
6. Meisner J, Goldberg JD. 2016. The Escherichia coli rhaSR-PrhaBAD Inducible Promoter System Allows Tightly Controlled Gene Expression over a Wide Range in Pseudomonas aeruginosa. [Appl Environ Microbiol](https://www.ncbi.nlm.nih.gov/pmc/articles/PMC5086565/). 82(22): 6715–6727. Published online 2016 Oct 27. Prepublished online 2016 Sep 9. doi: [10.1128/AEM.02041-16](https://dx.doi.org/10.1128%2FAEM.02041-16)
